# A Mobile Lifestyle Management Program (GlycoLeap) for People With Type 2 Diabetes: Single-Arm Feasibility Study

**DOI:** 10.2196/12965

**Published:** 2019-05-24

**Authors:** David Koot, Paul Soo Chye Goh, Robyn Su May Lim, Yubing Tian, Teng Yan Yau, Ngiap Chuan Tan, Eric Andrew Finkelstein

**Affiliations:** 1 SingHealth Polyclinics Singapore Singapore; 2 Health Services and Systems Research Duke-NUS Medical School Singapore Singapore; 3 KKT Technology Pte Ltd Singapore Singapore

**Keywords:** type 2 diabetes mellitus, self-management, mobile health, mHealth, mobile phone app, mobile apps, health coaching, blood glucose, single-arm feasibility study, RE-AIM

## Abstract

**Background:**

Singapore’s current prevalence of diabetes exceeds 13.6%. Although lifestyle modification can be effective for reducing the risks for complications of type 2 diabetes mellitus (T2DM), traditional lifestyle interventions are often difficult to administer in the primary care setting due to limited resources. Mobile health apps can address these limitations by offering low-cost, adaptable, and accessible platforms for disseminating lifestyle management interventions.

**Objective:**

Using the RE-AIM evaluation framework, this study assessed the potential effectiveness and feasibility of GlycoLeap, a mobile lifestyle management program for people with T2DM, as an add-on to standard care.

**Methods:**

This single-arm feasibility study recruited 100 patients with T2DM and glycated hemoglobin (HbA_1c_) levels of ≥7.5% from a single community health care facility in Singapore. All participants were given access to a 6-month mobile lifestyle management program, GlycoLeap, comprising online lessons and the Glyco mobile phone app with a health coaching feature. The GlycoLeap program was evaluated using 4 relevant dimensions of the RE-AIM framework: (1) reach (percentage who consented to participate out of all patients approached), (2) effectiveness (percentage point change in HbA_1c_ [primary outcome] and weight loss [secondary outcome]), (3) implementation (program engagement as assessed by various participatory metrics), and (4) maintenance (postintervention user satisfaction surveys to predict the sustainability of GlycoLeap). Participants were assessed at baseline and at follow-up (≥12 weeks after starting the intervention).

**Results:**

A total of 785 patients were approached of whom 104 consented to participate, placing the reach at 13.2%. Four were excluded after eligibility screening, and 100 patients were recruited. Program engagement (implementation) started out high but decreased with time for all evaluated components. Self-reported survey data suggest that participants monitored their blood glucose on more days in the past week at follow-up compared to baseline (*P*<.001) and reported positive changes to their diet due to app engagement (*P*<.001) (implementation). Primary outcome data were available for 83 participants. Statistically significant improvements were observed for HbA_1c_ (–1.3 percentage points, *P*<.001) with greater improvements for those who logged their weight more often (*P*=.007) (effectiveness). Participants also had a 2.3% reduction in baseline weight (*P*<.001) (effectiveness). User satisfaction was high with 74% (59/80) and 79% (63/80) of participants rating the app good or very good and claiming that they would probably or definitely recommend the app to others, respectively (maintenance).

**Conclusions:**

Although measures of program engagement decreased with time, clinically significant improvements in HbA_1c_ were achieved with the potential for broader implementation. However, we cannot rule out that these improvements were due to factors unrelated to GlycoLeap. Therefore, we would recommend evaluating the effectiveness and cost effectiveness of GlycoLeap using a randomized controlled trial of at least 12 months.

**Trial Registration:**

ClinicalTrials.gov NCT03091517; https://clinicaltrials.gov/ct2/show/NCT03091517 (Archived by WebCite at http://www.webcitation.org/77rNqhwRn)

## Introduction

### Background

The worldwide prevalence of diabetes has almost doubled over the past three decades and continues to rise [1]. Singapore’s current prevalence of diabetes exceeds 13.6% [2], and forecasts predict that without successful interventions, the lifetime risk of developing type 2 diabetes mellitus (T2DM) in Singapore will be 1 in 2 by 2050 [3].

Lifestyle modification can be highly effective at reducing the risks for complications of T2DM [4], yet traditional lifestyle interventions are often difficult to administer in the primary care setting due to limited resources and infrequent patient interaction with health care personnel. Mobile health (mHealth) apps can address these limitations by offering low-cost, highly adaptable, and easily accessible platforms for disseminating lifestyle management interventions. Effective T2DM apps stress skill building; self-efficacy; and frequent monitoring of blood glucose levels, weight, dietary intake, and physical activity [5-9]. Several diabetes management mHealth interventions also provide participants with some level of personalized communication with health professionals, including real-time feedback [10-16].

Given the rising health and cost implications of diabetes in Singapore, a comprehensive program that incorporates the most effective mHealth strategies and personalized health coaching offers a potentially scalable model to address Singapore’s diabetes epidemic. One potential program is GlycoLeap (Holmusk), a proprietary lifestyle management program for adults with T2DM. The GlycoLeap program, which was originally developed for use in Singapore, comprises two components: a comprehensive T2DM educational curriculum delivered through online lessons and the Glyco mobile phone app with a health coaching feature. The Glyco app enables users to log and monitor their blood glucose levels, weight, meals, and physical activity, which is captured via the mobile phone’s built-in pedometer. The app also serves as a vehicle for accredited dietitians, known as health coaches, to provide personalized feedback to participants on their progress and to present opportunities for improvement.

### Goal of the Study

The goal of this study was to assess the potential effectiveness and feasibility of the GlycoLeap program as an add-on to standard care using the RE-AIM evaluation framework [17,18]. If results are promising, a 2-arm randomized controlled trial aimed to test effectiveness and cost effectiveness will be recommended.

## Methods

### Research Design

This effort consisted of a 6-month (24-week), single-arm, preintervention (baseline) and follow-up evaluation. One hundred participants were recruited by a research coordinator from a single community health care facility, SingHealth Polyclinics–Tampines, in Singapore. All participants signed an informed consent to participate in a baseline and follow-up assessment that was conducted at least 12 weeks after starting the intervention and to allow for medical records abstraction. At the baseline assessment, participants completed a brief survey (Multimedia Appendix 1) that captured demographic information, diabetes status, and self-care activity information (from the Summary of Diabetes Self-Care Activities scale [19]). At the follow-up, participants completed a survey (Multimedia Appendix 2) that captured self-reported changes in diabetes self-care [19], dietary consumption, physical activity, program engagement, and user satisfaction. Glycated hemoglobin (hemoglobin A_1c_, or HbA_1c_) levels, medications, and weight were measured or obtained from medical records at baseline and follow-up.

Participants were eligible to participate if they (1) were aged 21 to 70 years, (2) had been medically diagnosed with T2DM as listed in electronic health records, (3) had an HbA_1c_ result of ≥7.5% within the past 2 months, (4) had a body mass index (BMI) of >23 kg/m^2^, (5) were not on insulin, and (6) owned and were able to use an iPhone or Android mobile phone. Participants were excluded if they (1) had cancer requiring treatment in the past 5 years, (2) had cardiovascular diseases (heart attack or cardiac procedure within the past 3 months), (3) had stroke or history of treatment for transient ischemic attacks in the past 3 months, (4) had chronic renal failure or were on dialysis, (5) had any amputation of lower limbs, (6) were using medication for weight loss, (7) had chronic treatment with systemic corticosteroids, (8) had bariatric surgery or extensive bowel resection, (9) were unable to converse in or read and write English, or (10) did not have a valid HbA_1c_ blood test within the 2 months prior to the date of recruitment. Each participant was compensated SGD20 (approximately US $15) in vouchers at study completion. All study procedures were approved by the SingHealth Centralized Institutional Review Board (CIRB Ref: 2017/2013) and the study is registered at ClinicalTrials.gov [NCT03091517].

### Intervention Program

All eligible participants received 24 weeks of free, unlimited access to the GlycoLeap program. Participants downloaded the Glyco app onto their mobile phones upon recruitment. An Accu-Chek Performa (F. Hoffmann–La Roche Ltd) glucometer kit with lancets and test strips, a BodyTrace (BodyTrace Inc) wireless weighing scale, and a resistance band for strength training were provided at no cost to participants. Although the recommendation to switch glucometers may have been an inconvenience, this was not raised as a concern by any of the participants. Participants were also given printed instruction guides on operating the Glyco app and devices and two guidebooks educating them on how to make healthier food choices and achieve weight loss. Table 1 describes key components of the GlycoLeap program and the recommended frequencies of engagement. Screenshots illustrating the Glyco app user interface are presented in Multimedia Appendices 3-7.

**Table 1:** Description of GlycoLeap program components and recommendations for engagement.

Component	Description	Recommended frequency
Online health lessons on diabetes and self-management	A total of 24 educational lessons on diabetes and self-management were delivered online. This curriculum was adapted for the local population and covers topics that take reference from the 7 healthy self-care behaviors as described by the American Association of Diabetes Educators. Quizzes tested knowledge on diabetes, obtained information about participants’ lifestyle habits, and were designed to keep participants engaged throughout each lesson.	Complete one lesson (lasting about 15 minutes) per week
Blood glucose monitoring	Blood glucose measurements obtained using the Accu-Chek Performa glucometer kit were input manually by participants into their Glyco app accounts.	At least 4 blood glucose logs per week (preferably paired pre- and postmeal readings)
Weight monitoring	Wireless weighing scale readings were automatically synced to participants’ accounts via cellular connectivity (3G).	At least one weight log per week
Meal logging	Meal photos taken by participants were uploaded onto the app for health coach evaluation. Health coaches rate meals using a 1 to 5 linear scale. Meal scores are awarded based on the balance of nutrients, food quality, and nutritional content. The meal scores take reference from the Singapore Health Promotion Board’s national dietary guidelines.	No recommendation was provided. Participants were encouraged to log as often as they wanted to.
Physical activity tracking	The Glyco app tracks the number of daily steps taken using the participants’ built-in phone pedometers.	70,000 steps per week
Health coach	Health coaches rate and respond to all meal logs and regularly send messages to participants to provide recommendations, encouragement, and personalized feedback on progress and answer participants’ questions (Multimedia Appendices 6 and 7). Correspondence is 2-way and participants are free to initiate or respond to messages from their health coach. All correspondence is conducted in-app, and all participants receive health coaching regardless of whether they send any messages to their health coach. Coaches capitalize on modalities that have been shown to lead to effective behavior change [20,21], including several theoretical frameworks [22-25], and are trained in the nutrition care process [26].	No recommendation was provided. Participants were encouraged to engage as often as they wanted to.

### Program Evaluation

The GlycoLeap program was evaluated using relevant dimensions of the RE-AIM framework [17,18].

#### Reach

A proxy for Reach was used and defined as the percentage of those who gave informed consent to participate out of all patients approached.

#### Effectiveness

As this was a feasibility study without a control group, measures of potential effectiveness were assessed as changes in HbA_1c_ levels (primary outcome) and weight (secondary outcome) between baseline and the follow-up. The analysis was conducted separately on the total sample with baseline values carried forward for those with missing data at follow-up (intention-to-treat [ITT] analysis) and on those who completed the study and did not initiate insulin (per-protocol analysis). All HbA_1c_ tests were conducted using the polyclinic’s protocols and approved laboratories, and weight was measured using validated weighing scales at the polyclinic. The window for eligible tests was defined as within 2 months before the scheduled baseline assessment and from 12 weeks to a maximum of 38 weeks after the intervention start date for the follow-up. In addition to the above indicators of potential effectiveness, the association between changes in health outcomes and measures of program engagement (as defined in the Implementation section) were evaluated using the per-protocol approach with the expectation that participants with greater levels of program engagement will show greater improvements in health outcomes.

#### Adoption

Adoption tends to focus more on system level factors and was not captured as part of this feasibility study.

#### Implementation

Implementation was assessed on the total sample by exploring engagement with key components of the GlycoLeap program as shown in Table 1. This includes the following process measures: (1) number of online health lessons completed, (2) number of blood glucose measurements logged per week, (3) number of weight measurements logged per week, (4) number of meal logs per week, and (5) number of messages sent by participants to health coaches per week. We did not evaluate results from the physical activity component because it would be an inaccurate reflection of activity given that participants were not expected to carry their mobile phones with them throughout the day and when exercising. We present the percentage of participants that engaged with the components over the intervention period. Self-reported changes in diabetes self-care activities and changes in lifestyle-related behaviors such as measures of dietary consumption and physical activity were assessed from survey responses. See Multimedia Appendices 1 and 2 for the baseline and follow-up surveys, respectively, which include the full list of self-reported measures.

#### Maintenance

Within-trial maintenance is included in the Implementation domain. Here, we assess sustainability at the setting level by exploring user satisfaction at follow-up (Multimedia Appendix 2) including ease of use, perceived value of each program component, an overall rating of the program, and whether or not participants would recommend the app to others. The idea is that the program has little chance of broader sustainability if it performs poorly in these measures.

### Sample Size and Statistical Analysis

Power calculations were not performed because this was a feasibility study. The target sample size of 100 was selected taking into consideration the aims of the study (testing acceptability and potential for effectiveness rather than efficacy) and practical feasibility. Studies on diabetes self-management mobile app interventions often involved a sample size of less than 100, including controlled trials [8,27].

Paired or one-sample *t* tests were conducted to assess for differences in means in HbA_1c_ and weight outcomes from baseline to follow-up. Associations between changes in health outcomes and measures of program engagement were assessed using linear regression, controlling for baseline health outcomes and for age, gender, and ethnicity. For all program engagement explanatory variables, participants were categorized into more engaged users if their total number of logs, messages, or lessons completed were above the median. Conversely, participants were categorized into less engaged users if their total number of logs, messages, or lessons completed were equal to or below the median. Analyses were performed on Stata/MP version 14.0 (StataCorp LLC) and R version 3.5.1 (The R Foundation).

## Results

### Reach and Sample Statistics

**Figure 1:**
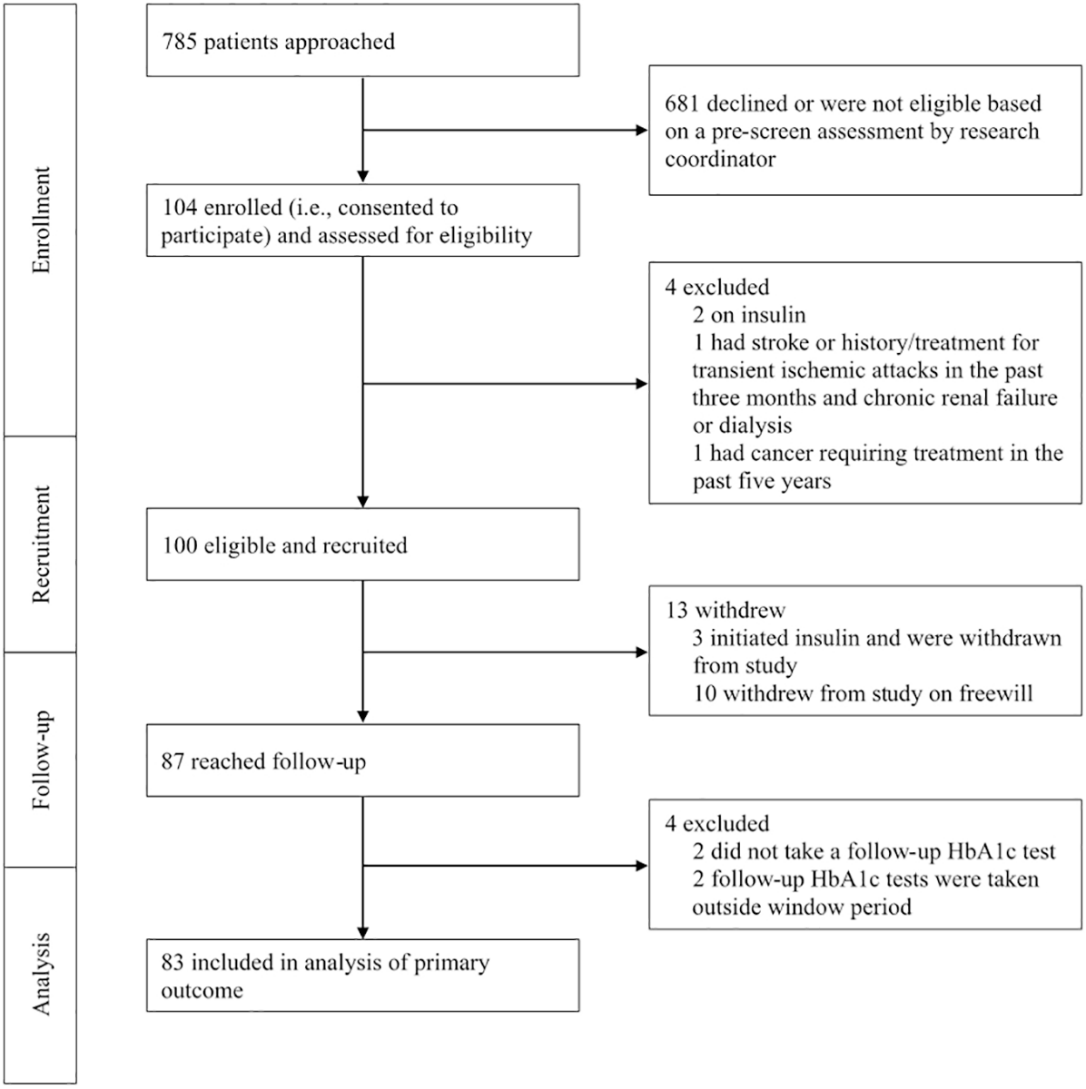
Participant recruitment and retention Consolidated Standards of Reporting Trials flow diagram.

Between June and November 2017, a total of 785 SingHealth Polyclinics–Tampines patients were approached by the research coordinator. Of these patients, 681 declined to participate or were deemed ineligible based on a prescreen assessment, placing the Reach at 13.2% (Figure 1). Although we did not record the reasons for prescreen failures, anecdotal information from the research coordinator suggests that the majority of patients were willing to participate but were assessed to be ineligible during prescreening due to insulin treatment, English illiteracy, or did not own a mobile phone. A total of 100 participants were recruited and all were given access to the 24-week GlycoLeap program.

The average age of participants was 54 years old, and 50 were male. Of the 100 participants, 45 were Chinese, 29 were Malay, 18 were Indian, and 8 were of other ethnicity. Sixty-one participants had high school-equivalent or lower education, and 69 were employed. At baseline, the mean HbA_1c_ and weight were 8.8% and 79.7 kg, respectively. On average, participants were diagnosed with T2DM 9.3 years ago. Other baseline characteristics can be found in Table 2.

Thirteen of the 100 participants withdrew from the study, either due to insulin initiation or free will (Figure 1). Of the 87 that reached follow-up, 4 were excluded from the per-protocol analysis as they either did not take a follow-up HbA_1c_ test or took their test outside the window (Figure 1). No statistically significant differences were found in baseline characteristics between the total sample (n=100, included in ITT analysis) and the completes (n=83, included in per-protocol analysis) (Table 2). Eighty of the 100 participants completed the follow-up survey.

**Table 2:** Baseline characteristics of participants in the single-arm GlycoLeap feasibility study.

Characteristics		Total (n=100)	Completes (n=83)	*P* value
Age (years), mean (SD)		53.5 (9.6)	53.5 (9.7)	.98
Male, n (%)		50 (50)	44 (53)	.67
Weight (kg), mean (SD)		79.7 (16.8)	79.2 (15.6)	.87
Height (cm), mean (SD)		163.1 (8.9)	163.5 (9.3)	.75
Body mass index (kg/m^2^), mean (SD)		29.8 (5.0)	29.5 (4.6)	.68
Systolic blood pressure (mm Hg), mean (SD)		132.1 (11.6)	132.5 (11.8)	.83
Diastolic blood pressure (mm Hg), mean (SD)		74.7 (10.6)	74.9 (10.5)	.88
HbA_1c_^a^ (%), mean (SD)		8.8 (1.6)	8.9 (1.7)	.96
Years diagnosed with diabetes, mean (SD)		9.3 (7.3)	8.8 (6.3)	.56
On oral medication for diabetes treatment, n (%)		98 (98)	81 (98)	.85
**Ethnicity, n (%)**				**.86**
	Chinese	45 (45)	38 (46)	—
	Malay	29 (29)	22 (27)	—
	Indian	18 (18)	15 (18)	—
	Other	8 (8)	8 (10)	—
**Highest completed education level, n (%)**				**.71**
	High school or lower	61 (61)	49 (59)	—
	Precollege (A-levels/polytechnic diploma)	21 (21)	17 (20)	—
	College graduate/postgraduate	18 (18)	17 (20)	—
**Current marital status, n (%)**				**>.99**
	Never married	12 (12)	10 (12)	—
	Married	82 (82)	68 (82)	—
	Other	6 (6)	5 (6)	—
**Monthly household income, n (%)**				**.58**
	Less than SGD 5000	10 (10)	6 (7)	—
	SGD 5000-SGD 9999	2 (2)	2 (2)	—
	≥SGD 10,000	1 (1)	1 (1)	—
	Prefer not to say	87 (87)	74 (89)	—
**Employment status, n (%)**				**.78**
	Working (full-/part-time)	69 (69)	59 (71)	—
	Homemaker^b^	22 (22)	17 (20)	—
	Retired and not working^c^	9 (9)	7 (8)	—

^a^HbA_1c_: glycated hemoglobin.

^b^Individuals who are full-time housekeepers, regardless of prior employment status.

^c^Individuals who were previously employed until retirement and are typically past retirement age.

### Implementation (Program Engagement)

**Figure 2:**
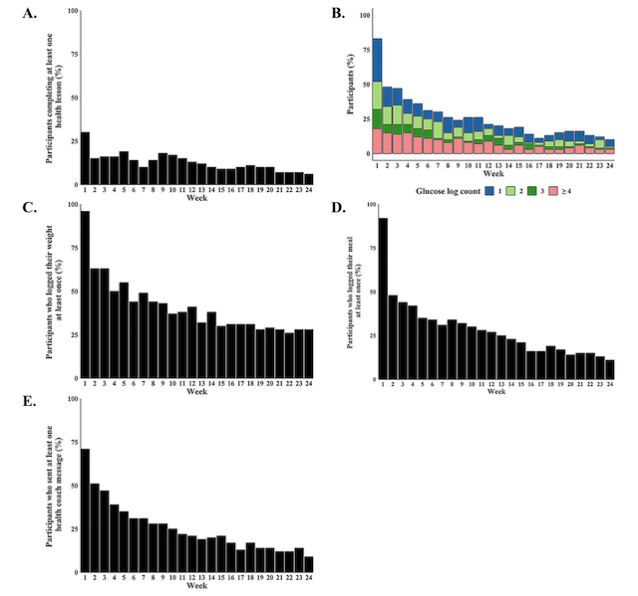
Proportion of program engagement by week for the total sample (n=100). Percentage of participants who (A) completed at least one lesson, (B) logged 1, 2, 3, or ≥4 glucose measurements, (C) logged at least one weight log, (D) logged at least one meal log, or (E) sent at least one message to their health coach a week.

Generally high participant engagement was observed for all components in the first week which then decreased progressively over time. Trends in program engagement for the total sample (n=100) are shown in Figure 2. At the end of the 24-week program, a third of the participants (33/100, 33.0%) completed at least one online health lesson. On average, participants finished 9.2 lessons. Throughout the intervention period, fewer than 20 participants logged their blood glucose measurements at least 4 times a week yet more than 25 participants logged their weight measurements at least once a week, which were the recommendations. On average, participants entered 2.1 meal logs and sent 2.8 messages to their health coach each week. The mean, median, minimum, and maximum total number of messages sent by participants to their health coaches throughout the 24-week program were 66.9, 8.5, 0, and 799, respectively. For total number of messages sent by health coaches to participants throughout the entire study period, the mean, median, minimum, and maximum were 234.9, 69, 16, and 2754, respectively.

In total, two of the 100 participants did not engage with any of the 5 evaluated components, and 14 engaged with at least one component every week throughout the intervention period. Thirteen participants engaged with the same component(s) every week throughout the intervention period: health lessons (2 participants), blood glucose monitoring (3 participants), weight monitoring (6 participants), meal logging (7 participants), and health coach messaging (5 participants).

Self-reported changes in diabetes self-care activities and lifestyle behaviors at follow-up from baseline are show in Table 3 for the 80 participants who completed the follow-up survey. Participants reported monitoring their blood glucose on more days in the week before the follow-up assessment compared to the week before starting the intervention (2.3 days [95% CI 1.9-2.7] vs 0.6 days [95% CI 0.2-1.0], *P*<.001). Similarly, 68 out of 80 participants (85%, *P*<.001) declared positive changes in diet due to app engagement. On average, participants reported consuming the recommended servings of fruit and vegetables on more days in the past week at follow-up compared to baseline (3.7 days [95% CI 3.1-4.2] vs 1.3 days [95% CI 0.8-1.8], *P*<.001) and high fat food on fewer days (1.6 days [95% CI 1.2-2.0] vs 2.3 days [95% CI 1.9-2.7], *P*=.003). Although 30 out of 80 participants (38%) claimed to have increased their weekly average level of moderate-to-vigorous physical activity due to app engagement, there was no statistically significant difference in the number of days where participants reported performing at least 30 minutes of continuous activity in the past week at follow-up compared to baseline (3.9 days [95% CI 3.4-4.4] vs 3.4 days [95% CI 2.7-4.0], *P*=.14).

**Table 3:** Self-reported diabetes self-care activities and lifestyle behaviors at baseline and follow-up (n=80).

Category and behavior or activity		Baseline	Follow-up	Difference	*P* value
**Blood glucose monitoring, mean (95% CI)**					
	Days that blood glucose was monitored in past week	0.6 (0.2 to 1.0)	2.3 (1.9 to 2.7)	1.7 (1.3 to 2.1)	<.001
**Dietary habits**					
	Positive change in diet due to app engagement, n (%)	—^a^	68 (85)	N/A^b^	<.001
	Days with fruit and vegetable consumption as per recommended servings in past week, mean (95% CI)	1.3 (0.8 to 1.8)	3.7 (3.1 to 4.2)	2.4 (1.6 to 3.1)	<.001
	Days with high fat food consumption in past week, mean (95% CI)	2.3 (1.9 to 2.7)	1.6 (1.2 to 2.0)	–0.7 (–1.1 to –0.2)	.003
**Physical activity, mean (95% CI)**					
	Days with at least 30 minutes of continuous activity including walking in past week	3.4 (2.7 to 4.0)	3.9 (3.4 to 4.4)	0.5 (–0.2 to 1.2)	.14
**Change in average weekly level of moderate-to-vigorous physical activity due to app engagement, n (%)**					**<.001**
	Increased	—	30 (38)	N/A	
	Decreased	—	0 (0)	N/A	
	Stayed the same	—	50 (63)	N/A	

^a^Question was not present in the baseline survey as it asks for self-reported change due to app engagement.

^b^N/A: not applicable as question was not present in the baseline survey.

### Effectiveness (Health Outcomes)

**Figure 3:**
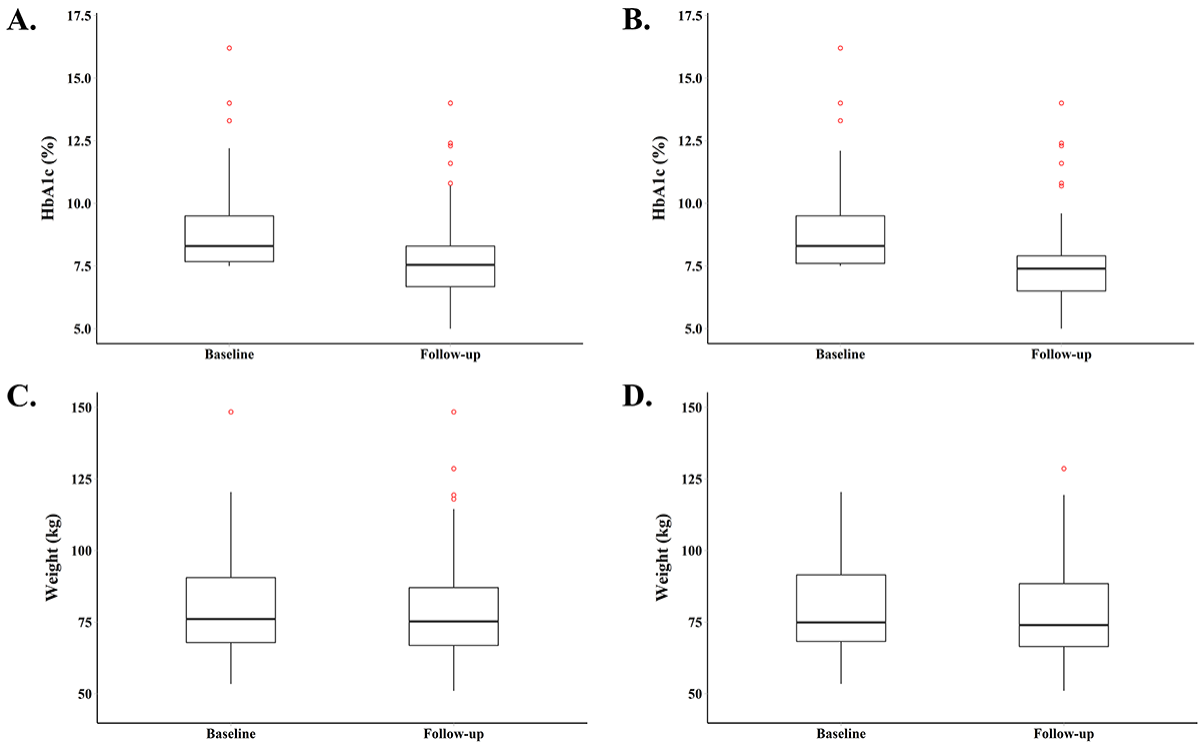
Distributions of health outcomes at baseline and follow-up: (A) HbA_1c_ for total sample (n=100), (B) HbA_1c_ for completes (n=83), (C) weight for total sample (n=100), and (D) weight for completes (n=83).

Changes in HbA_1c_ levels and weight are shown in Figure 3 and Table 4. Per-protocol analysis revealed that, on average, participants’ HbA_1c_ levels were 1.3 percentage points lower at follow-up compared to baseline (7.6% [95% CI 7.2-7.9] vs 8.9% [95% CI 8.5-9.2], *P*<.001) and 49 of 83 participants (59%) achieved a ≥1 percentage point reduction in HbA_1c_ levels. The average duration between intervention start and follow-up HbA_1c_ tests was 24.2 weeks. Based on per-protocol analysis, participants achieved a weight loss of 2.3 kg at follow-up compared to baseline (77.3 kg [95% CI 74.0-80.7] vs 79.2 kg [95% CI 75.8-82.6], *P*<.001), and 17 out of 83 participants (20%) lost ≥5% of their initial body weight at baseline. Similar results and levels of statistical significance were obtained with the ITT analysis (Table 4).

Using the per-protocol approach, linear regression showed that HbA_1c_ decreased by an average of 1.0 percentage point more among those who logged their weight more (*P*=.007) (Multimedia Appendix 8). No statistically significant associations were found between change in weight and measures of program engagement (data not shown).

**Table 4:** Changes in health outcomes at follow-up compared to baseline for participants who reached follow-up.

Health outcomes and measures		Total (n=100)	*P* value	Completes (n=83)	*P* value
**HbA_1c_^a^ change**					
	Percentage point change in HbA_1c_, mean (95% CI)	–1.1 (–1.4 to –0.7)	<.001	–1.3 (–1.7 to –0.8)	<.001
	Participants with ≥1 percentage point reduction, n (%)	49 (49)		49 (59)	
**Weight change**					
	Weight change expressed as a percentage of baseline weight (%), mean (95% CI)	–2.0 (–2.8 to –1.2)	<.001	–2.3 (–3.3 to –1.4)	<.001
	Participants with loss of ≥5% of initial baseline weight, n (%)	17 (17)		17 (21)	

^a^HbA_1c_: glycated hemoglobin.

### Maintenance (User Satisfaction)

Among the 80 participants who completed the follow-up survey, the average user-friendliness rating for various program components was 3.6 to 3.8 out of 5, where 5 = very easy (Multimedia Appendix 9). Program components received perceived usefulness scores of 3.3 to 3.5 out of 5, where 5 = very useful (Multimedia Appendix 10). Despite being given new glucometers to use for the study, participants gave average ratings of 3.8 and 3.5 out of 5 for glucometer user-friendliness and usefulness of performing blood glucose monitoring, respectively (Multimedia Appendices 9 and 10). Most participants gave the Glyco app an overall rating of good (41/80, 51%) or very good (18/80, 23%) (Multimedia Appendix 11). The majority of participants indicated that they either probably would (42/80, 53%) or definitely would (21/80, 26%) recommend the Glyco app to others for managing their diabetes. Only one participant probably would not recommend the app to others, and 20% (16/80) were neutral on this matter. Respondent data revealed that 21% (17/80) of participants stated that they were willing to purchase unlimited access to the Glyco app and health coaches for an annual fee of SGD100 (approximately US $73) but no participant said that they were willing to pay an annual fee of SGD200 (approximately US $147).

## Discussion

### Principal Findings

A reduction in HbA_1c_ levels of 1 percentage point has been shown to be associated with a 21%, 14%, 37%, and 21% decrease in risk of any end point related to diabetes, myocardial infarction, microvascular complications, and diabetes-related death, respectively [28]. This study found that on average, participants achieved this level of improvement in HbA_1c_ level over the study period. Feasibility studies using mobile phone–based lifestyle management interventions for diabetes typically do not observe such large changes [9,11,29,30]. Additionally, almost a fifth of participants—all of whom were overweight (BMI >23 kg/m^2^) at baseline—lost more than 5% of their initial body weight. Behavioral interventions have demonstrated that a weight loss of as little as 5% among overweight adults can result in health benefits [31]. Overall, these results are encouraging and suggest that the mHealth app has the potential to be clinically relevant in practice. However, we cannot rule out the possibility that factors unrelated to GlycoLeap could be positively influencing health outcomes.

### Strengths and Limitations

In spite of relatively high levels of engagement across Glyco app components in the first week, the decreased usage over time advocates that more should be done to improve and sustain engagement. Nevertheless, despite low completion rates for the online lessons, self-reported increases in blood glucose monitoring frequency and improvements in dietary habits argue that a mobile intervention like GlycoLeap may be a viable strategy for patient education and behavior change. As the online lessons were administered on a different platform and required separate email access, this may have presented a barrier to access. A greater completion rate may have been achieved if the lessons were made available directly on the mobile app.

Unlike conventional T2DM lifestyle management programs administered in the primary care setting through in-person sessions, mHealth interventions comprising mobile phone apps like Glyco are highly scalable, requiring comparatively fewer manpower resources. Although we were unable to determine the true reach as defined by the RE-AIM framework [17,18], we attempted to obtain a proxy for reach at SingHealth Polyclinics–Tampines using the percentage of patients who consented to participate out of all patients approached. Despite an apparent low reach of 13.2%, this percentage does not accurately represent interest in GlycoLeap as patients who were deemed ineligible (eg, unable to speak English or did not have a mobile phone) during the prescreen assessment were not offered the opportunity to give informed consent and take the screener. Based on the anecdotal information from the research coordinator, most of the patients approached were keen to join the study. Hence, we anticipate that the actual willingness to participate in GlycoLeap if offered as an add-on to standard care will be higher than 13.2%, especially if GlycoLeap is made available in other languages such as Mandarin and mobile phone use becomes more prevalent.

This study also suggests that GlycoLeap might be a scalable intervention. The app received good ratings with 21% (17/80) of participants claiming they were willing to purchase unlimited access to use the app and health coaches at a modest fee. In addition, the GlycoLeap program received relatively high user-friendliness and user-satisfaction scores compared to other similar mobile phone apps for diabetes management [9,32].

### Conclusions

These results suggest that GlycoLeap may be an effective strategy for helping some adults with T2DM attain better diabetic control and that it is feasible to integrate it within the primary care setting. However, given the study design, we cannot exclude the possibility that any health improvements were due to factors unrelated to the GlycoLeap program. Therefore, future efforts should assess the effectiveness and cost effectiveness of GlycoLeap using a randomized controlled trial of at least 12 months to evaluate longer term outcomes.

## Appendix

Multimedia Appendix 1 Baseline survey.

Multimedia Appendix 2 Follow-up survey.

Multimedia Appendix 3 Glyco app home screen.

Multimedia Appendix 4 Glyco app blood glucose monitoring.

Multimedia Appendix 5 Glyco app weight monitoring.

Multimedia Appendix 6 Glyco app meal log.

Multimedia Appendix 7 Glyco app health coach correspondence.

Multimedia Appendix 8 Output of linear regression with change in HbA_1c_ regressed on measures of program engagement.

Multimedia Appendix 9 Average participant rating of the user-friendliness of different GlycoLeap program components, where 1=very difficult and 5=very easy.

Multimedia Appendix 10 Average participant rating of the perceived usefulness of different GlycoLeap program components, where 1=not useful at all and 5=very useful.

Multimedia Appendix 11 Distribution of overall rating of the Glyco app, where 1=very poor, 2=poor, 3=average, 4=good, and 5=very good.

## Abbreviations

BMI: body mass index
HbA_1c_: hemoglobin A_1c_ (glycated hemoglobin)
ITT: intention-to-treat
mHealth: mobile health
T2DM: type 2 diabetes mellitus
