# Supplementary material for: A Mobile Lifestyle Management Program (GlycoLeap) for People With Type 2 Diabetes: Single-Arm Feasibility Study
Source: JMIR Mhealth Uhealth. 2019 May 24;7(5):e12965. doi: 10.2196/12965 (PMC6555118; doi:10.2196/12965)
Supplement: Multimedia Appendix 1 [file mhealth_v7i5e12965_app1.pdf]

Baseline survey

SECTION 1: SOCIO-DEMOGRAPHIC QUESTIONS

1. How old were you on your last birthday?
   ________ years

2. Are you ________?
   ☐Male
   ☐Female

3. Which ethnic group do you belong to?
   ☐Chinese
   ☐Malay
   ☐Indian
   ☐Other (please specify: ________________)

4. What is your current marital status?
   ☐Never married
   ☐Married
   ☐Widowed
   ☐Divorced
   ☐Separated from spouse
   ☐Prefer not to say

5. What is the highest level of education you have completed?
   ☐No formal education
   ☐Primary
   ☐Secondary
   ☐'A' level certificate
   ☐Polytechnic diploma
   ☐University first degree
   ☐University postgraduate diploma/degree
   ☐Other (please specify: ________________)

6. What is your household's total income from all sources, after taxes and compulsory deductions?
   ☐Below $1,000
   ☐$1,000 - $1,999
   ☐$2,000 - $2,999
   ☐$3,000 - $3,999
   ☐$4,000 - $4,999
   ☐$5,000 - $5,999
   ☐$6,000 - $6,999
   ☐$7,000 - $7,999
   ☐$10,000 and over
   ☐Prefer not to say

7. Which of the following best describes your current employment status?
 Working (full-time)
 Working (part-time)
 Homemaker
 Retired and not working
 Currently not working

SECTION 2: DIABETES STATUS

8. How many years back did your doctor diagnose you with type 2 diabetes?
________ years back

9. What is your current medication regimen for treatment of diabetes?
 No medication
 1 oral medication
 2 or more oral medications

SECTION 3: SUMMARY OF DIABETES SELF-CARE ACTIVITIES

10. How many of the last **seven days** have you followed a healthful eating plan?
 0
 1
 2
 3
 4
 5
 6
 7

11. On average, over the past month, how many **days per week** have you followed your eating plan?
 0
 1
 2
 3
 4
 5
 6
 7

12. On how many of the last **seven days** did you eat five or more servings of fruits and vegetables?
    - ☐0
    - ☐1
    - ☐2
    - ☐3
    - ☐4
    - ☐5
    - ☐6
    - ☐7

13. On how many of the last **seven days** did you eat high fat foods such as red meat or full-fat dairy products?
    - ☐0
    - ☐1
    - ☐2
    - ☐3
    - ☐4
    - ☐5
    - ☐6
    - ☐7

14. On how many of the last **seven days** did you participate in at least 30 minutes of physical activity (total minutes of continuous activity, including walking)?
    - ☐0
    - ☐1
    - ☐2
    - ☐3
    - ☐4
    - ☐5
    - ☐6
    - ☐7

15. On how many of the last **seven days** did you participate in a specific exercise session (such as swimming, walking, biking) other than what you do around the house or as part of your work?
    - ☐0
    - ☐1
    - ☐2
    - ☐3
    - ☐4
    - ☐5
    - ☐6
    - ☐7

16. On how many of the last **seven days** did you test your blood sugar?
   □0
   □1
   □2
   □3
   □4
   □5
   □6
   □7

17. On how many of the last **seven days** did you test your blood sugar the number of times recommended by your healthcare provider?
   □0
   □1
   □2
   □3
   □4
   □5
   □6
   □7

18. On how many of the last **seven days** did you test check your feet?
   □0
   □1
   □2
   □3
   □4
   □5
   □6
   □7

19. On how many of the last **seven days** did you inspect the inside of your shoes?
   □0
   □1
   □2
   □3
   □4
   □5
   □6
   □7

20. Have you smoked a cigarette – even one puff – during the past **seven days**?
   □Yes
   □No
   {If 'Yes', proceed to Q21, else skip to end of survey}

21. How many cigarettes did you smoke on an average day?

   ____
