# Supplementary material for: A Mobile Lifestyle Management Program (GlycoLeap) for People With Type 2 Diabetes: Single-Arm Feasibility Study
Source: JMIR Mhealth Uhealth. 2019 May 24;7(5):e12965. doi: 10.2196/12965 (PMC6555118; doi:10.2196/12965)
Supplement: Multimedia Appendix 2 [file mhealth_v7i5e12965_app2.pdf]

<u>Follow-up survey</u>

SECTION 1: DIABETES STATUS AND RELATED BEHAVIORS

1. What is your current medication regimen for treatment of diabetes?
   □No medication
   □Oral medication
   □Injectable medication

SECTION 2: SUMMARY OF DIABETES SELF-CARE ACTIVITIES

2. How many of the last **seven days** have you followed a healthful eating plan?
   □0
   □1
   □2
   □3
   □4
   □5
   □6
   □7

3. On average, over the past month, how many **days per week** have you followed your eating plan?
   □0
   □1
   □2
   □3
   □4
   □5
   □6
   □7

4. On how many of the last **seven days** did you eat five or more servings of fruits and vegetables?
   □0
   □1
   □2
   □3
   □4
   □5
   □6
   □7

5. On how many of the last **seven days** did you eat high fat foods such as red meat or full-fat dairy products?
   □0
   □1
   □2
   □3
   □4
   □5
   □6
   □7

6. Did you make any changes to your diet over the past six months as a result of using the Glyco app?
   □Yes
   □No
   {If 'Yes', proceed to Q7, else skip to Q8}

7. Please indicate what are the changes that you have made to your diet over the past six months as a result of using the *Glyco* app?

8. Did your average weekly level of <u>moderate to vigorous</u> physical activity change over the past six months, as a result of using the *Glyco* app?
   (Moderate or vigorous activity is defined as any activity that produces a small to large increase in breathing or heart rate, lasting for **at least 30minutes**)
   □Increased
   □Decreased
   □Stayed the same

9. On how many of the last **seven days** did you participate in at least 30 minutes of physical activity (total minutes of continuous activity, including walking)?
   □0
   □1
   □2
   □3
   □4
   □5
   □6
   □7

10. On how many of the last **seven days** did you participate in at least 30 minutes of <u>moderate to vigorous</u> physical activities? (Moderate or vigorous activity is defined as any activity that produces a small to large increase in breathing or heart rate, lasting for **at least 30minutes**)
    □0
    □1
    □2
    □3
    □4
    □5
    □6
    □7

11. On how many of the last **seven days** did you participate in a specific exercise session (such as swimming, walking, biking) other than what you do around the house or as part of your work?
    □0
    □1
    □2
    □3
    □4
    □5
    □6
    □7

12. Did you use the resistance band that was given to you, as part of the *GlycoLeap* program?
    □Yes
    □No
    {If 'Yes', proceed to Q13, else skip to Q14}

13. How many times in the last **seven days** did you use your resistance bands to perform any strength training exercises?
    □0
    □1
    □2
    □3 or more

14. On how many of the last **seven days** did you test your blood sugar?
    □0
    □1
    □2
    □3
    □4
    □5
    □6
    □7

15. On how many of the last **seven days** did you test check your feet?
   0
   1
   2
   3
   4
   5
   6
   7

16. On how many of the last **seven days** did you inspect the inside of your shoes?
   0
   1
   2
   3
   4
   5
   6
   7

17. Have you smoked a cigarette – even one puff – during the past **seven days**?
   Yes
   No
   {If 'Yes', proceed to Q18, else skip to Q19}

18. How many cigarettes did you smoke on an average day?
   ____

19. Did you chat with your health coach through the *Glyco* app?
   Yes
   No
   {If 'Yes', proceed to Q20, else skip to Q21}

20. To what extent have you followed the advice provided to you by the *Glyco* app health coaches?
   Not at all
   To a small extent
   To a moderate extent
   To a large extent

SECTION 3: PROGRAM EVALUATION

21. How user-friendly would you score each component of the *GlycoLeap* program?
    (Please score out of 5: 1 = Very difficult, 5 = Very easy)
    - Accessing weekly health lessons score: ________ (out of 5)
    - Operating the glucometer score: ________ (out of 5)
    - Logging blood sugar readings on the *Glyco* app score: ________ (out of 5)
    - Syncing measurements from wireless weighing scale to the *Glyco* app score: ________ (out of 5)
    - Logging daily meals on the *Glyco* app score: ________ (out of 5)
    - Tracking physical activity on the *Glyco* app score: ________ (out of 5)
    - Communicating with health coach on the *Glyco* app score: ________ (out of 5)

22. How useful would you score each feature of the *Glyco* app in helping you control your diabetes progression or improving your lifestyle habits?
    (Please score out of 5: 1 = Not Useful at all, 5 = Very Useful)
    - Health lessons score: ________ (out of 5)
    - Glucose monitoring score: ________ (out of 5)
    - Weight monitoring score: ________ (out of 5)
    - Meal logging score: ________ (out of 5)
    - Physical activity tracking score: ________ (out of 5)
    - Interaction with health coach score: ________ (out of 5)

23. Which, if any, of these problems did you face throughout your experience with the *GlycoLeap* program? (Check all the boxes that apply to your experience)
    ☐Unable to gain timely assistance to troubleshoot/resolve issues with app
    ☐Trouble logging into the *GlycoLeap* personal account
    ☐App features are too difficult to navigate/use
    ☐Glucometer faulty/does not consistently work well)
    ☐Wireless weighing scale faulty/does not consistently work well)
    ☐App was unable to track my true level of physical activity accurately
    ☐Health coaches were unable to provide me with adequate guidance
    ☐Too little reminders were sent to encourage engagement with app
    ☐Too many reminders were sent to encourage engagement with app
    ☐Using the app took up too much of my time
    ☐I did not face any of these problems

24. What is your overall rating of the app?
    (Please score out of 5: 1= Very poor, 5 = Very good)
    Score: ________ (out of 5)

25. Would you recommend the *Glyco* app to other people to use for managing their diabetes?
    (Please score out of 5: 1 = Would not recommend, 5 = Would highly recommend)
    Score: ________ (out of 5)

26. If it costs $100 per year for unlimited access to the *Glyco* app and health coach(es), would you pay?
    - ☐Yes
    - ☐No

    {If yes, proceed to Q27, else skip to Q28}

27. If it costs $200 per year for unlimited access to the *Glyco* app, would you pay for access?
    - ☐Yes
    - ☐No

28. Are there any issues that you would like the study team to note, which could have improved your experience throughout this study?
