# Supplementary material for: A Mobile Lifestyle Management Program (GlycoLeap) for People With Type 2 Diabetes: Single-Arm Feasibility Study
Source: JMIR Mhealth Uhealth. 2019 May 24;7(5):e12965. doi: 10.2196/12965 (PMC6555118; doi:10.2196/12965)
Supplement: Multimedia Appendix 3 [file mhealth_v7i5e12965_app3.pdf]

●●●○○ SingTel 100%

Weight

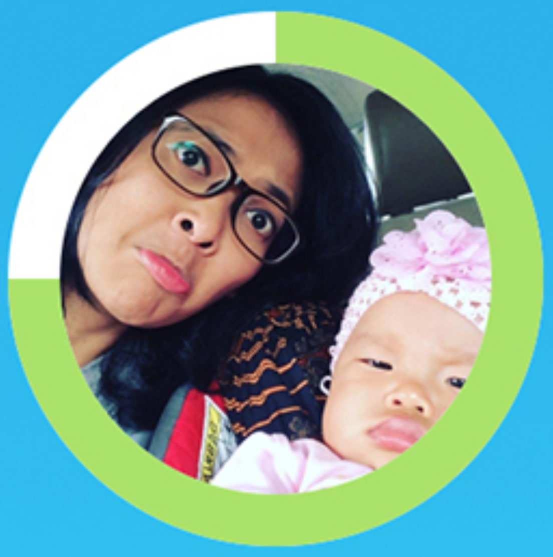

CURRENT

87.3

KG

START	GOAL
**90.3**	**72.0**
KG	KG

ALL FOOD GLUCOSE WEIGHT

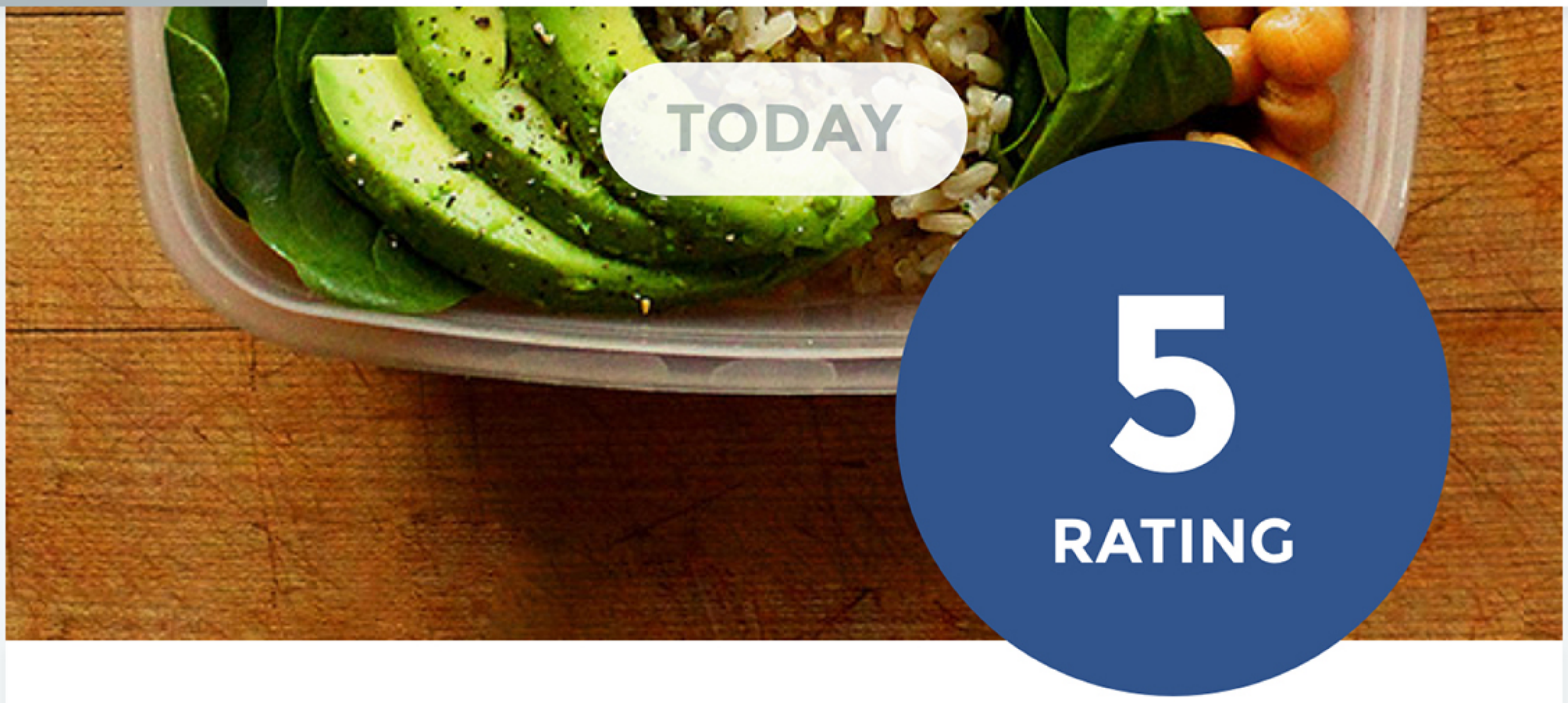

brown Rice, peas, spin..

Feed Ask Coach Insight Progress
