# Supplementary material for: A Mobile Lifestyle Management Program (GlycoLeap) for People With Type 2 Diabetes: Single-Arm Feasibility Study
Source: JMIR Mhealth Uhealth. 2019 May 24;7(5):e12965. doi: 10.2196/12965 (PMC6555118; doi:10.2196/12965)
Supplement: Multimedia Appendix 4 [file mhealth_v7i5e12965_app4.pdf]

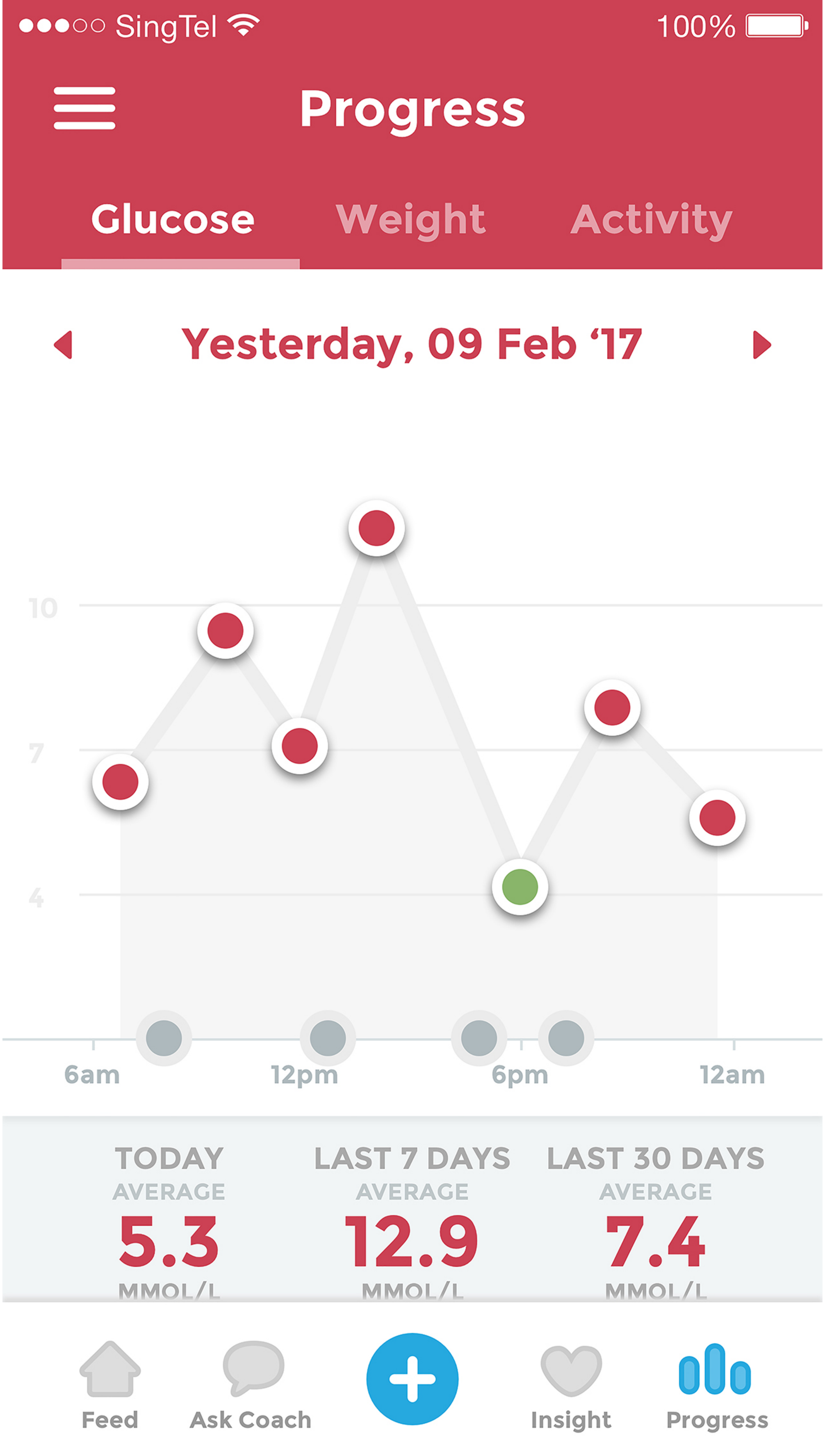
SingTel
100%
Progress
Glucose
Weight
Activity
Yesterday, 09 Feb '17
10
7
4
6am
12pm
6pm
12am
TODAY
AVERAGE
5.3
MMOL/L
LAST 7 DAYS
AVERAGE
12.9
MMOL/L
LAST 30 DAYS
AVERAGE
7.4
MMOL/L
Feed
Ask Coach
Insight
Progress
