# Supplementary material for: A Mobile Lifestyle Management Program (GlycoLeap) for People With Type 2 Diabetes: Single-Arm Feasibility Study
Source: JMIR Mhealth Uhealth. 2019 May 24;7(5):e12965. doi: 10.2196/12965 (PMC6555118; doi:10.2196/12965)
Supplement: Multimedia Appendix 5 [file mhealth_v7i5e12965_app5.pdf]

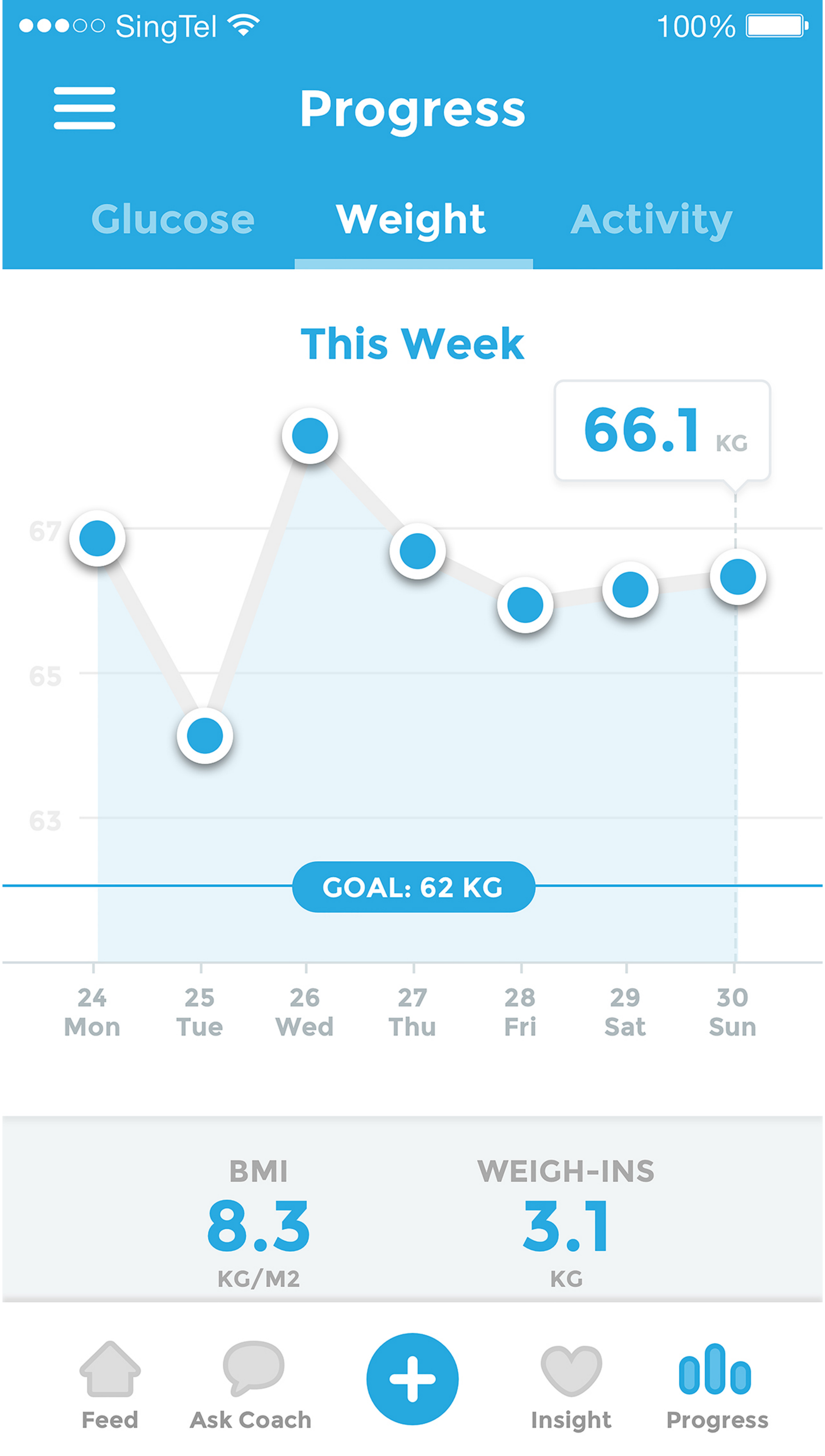
SingTel
100%
Progress
Glucose
Weight
Activity
This Week
66.1 KG
67
65
63
GOAL: 62 KG
24 Mon
25 Tue
26 Wed
27 Thu
28 Fri
29 Sat
30 Sun
BMI
8.3
KG/M2
WEIGH-INS
3.1
KG
Feed
Ask Coach
Insight
Progress
