# Supplementary material for: A Mobile Lifestyle Management Program (GlycoLeap) for People With Type 2 Diabetes: Single-Arm Feasibility Study
Source: JMIR Mhealth Uhealth. 2019 May 24;7(5):e12965. doi: 10.2196/12965 (PMC6555118; doi:10.2196/12965)
Supplement: Multimedia Appendix 6 [file mhealth_v7i5e12965_app6.pdf]

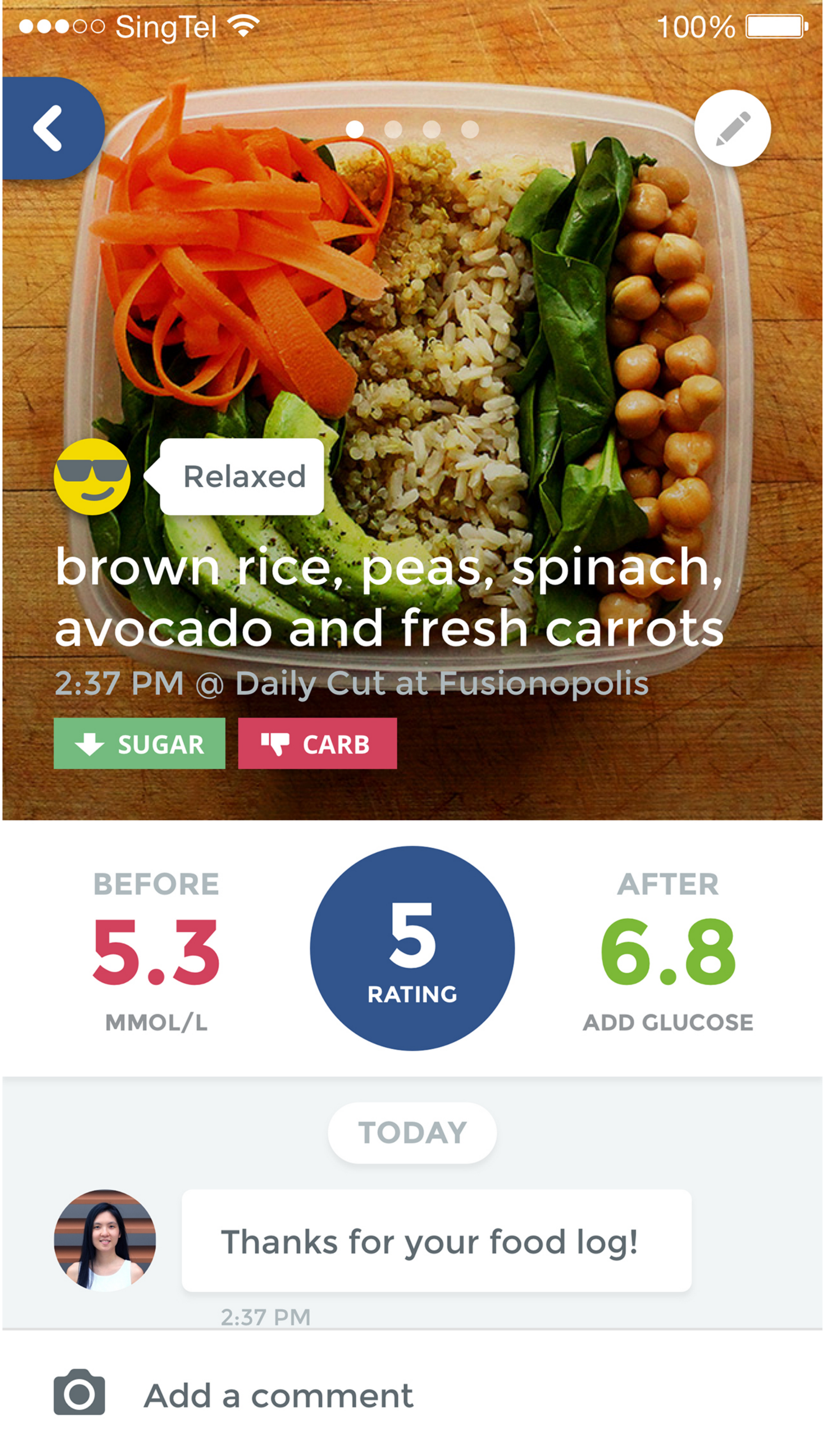
SingTel
100%
Relaxed
brown rice, peas, spinach, avocado and fresh carrots
2:37 PM @ Daily Cut at Fusionopolis
SUGAR
CARB
BEFORE
5.3
MMOL/L
5
RATING
AFTER
6.8
ADD GLUCOSE
TODAY
Thanks for your food log!
2:37 PM
Add a comment
