# Supplementary material for: A Mobile Lifestyle Management Program (GlycoLeap) for People With Type 2 Diabetes: Single-Arm Feasibility Study
Source: JMIR Mhealth Uhealth. 2019 May 24;7(5):e12965. doi: 10.2196/12965 (PMC6555118; doi:10.2196/12965)
Supplement: Multimedia Appendix 7 [file mhealth_v7i5e12965_app7.pdf]

●●●○○ SingTel 100%

brown rice, peas, spinach, avocado and fresh carrots

TODAY

Thanks for your food log!

2:37 PM

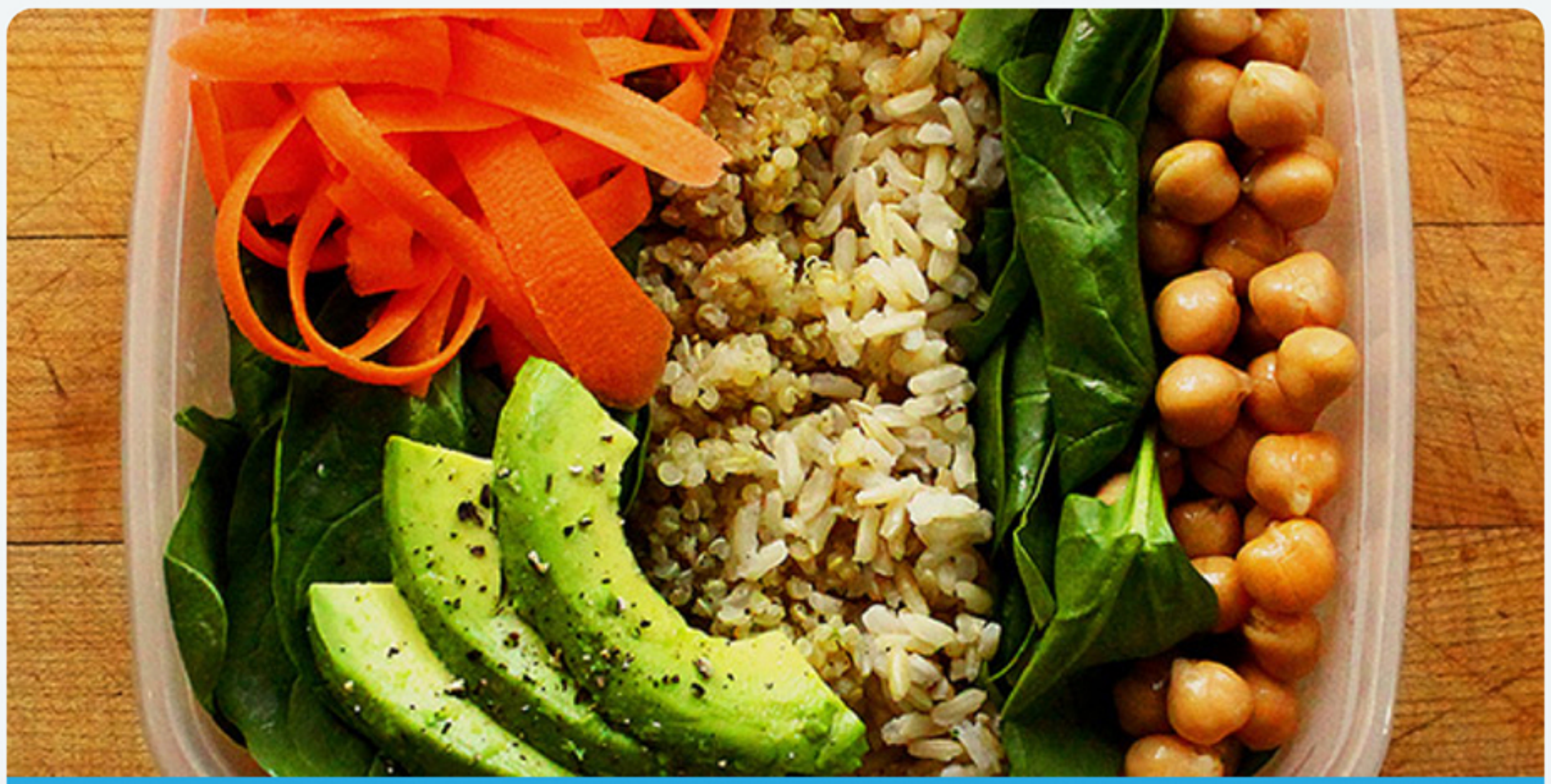

Yes I took the skin off!

4:52 PM

You're a champion! 👍

5:02 PM

But I also had corn. Not sure how much more I could take.

Add a comment
