# Supplementary material for: A Mobile Lifestyle Management Program (GlycoLeap) for People With Type 2 Diabetes: Single-Arm Feasibility Study
Source: JMIR Mhealth Uhealth. 2019 May 24;7(5):e12965. doi: 10.2196/12965 (PMC6555118; doi:10.2196/12965)
Supplement: Multimedia Appendix 8 [file mhealth_v7i5e12965_app8.pdf]

	Change in HbA1c
	Completes
Health Lessons	-0.180
	(0.373)
Glucose Logs	0.416
	(0.503)
Weight Logs	-0.995**
	(0.356)
Meal Logs	-0.988
	(0.555)
Health Coach Messages	-0.108
	(0.473)
Baseline HbA1c	-0.822***
	(0.105)
Age	-0.017
	(0.017)
Male	-0.035
	(0.310)
Chinese	-0.378
	(0.561)
Malay	-0.372
	(0.613)
Indian	0.014
	(0.648)
Constant	8.135***
	(1.664)
Observations	83
Adjusted R^2	0.536
Note:	*p<0.05; **p<0.01; ***p<0.001
