# Supplementary figures and images for: A Mobile Lifestyle Management Program (GlycoLeap) for People With Type 2 Diabetes: Single-Arm Feasibility Study
Source: JMIR Mhealth Uhealth. 2019 May 24;7(5):e12965. doi: 10.2196/12965 (PMC6555118; doi:10.2196/12965)

**Program components**

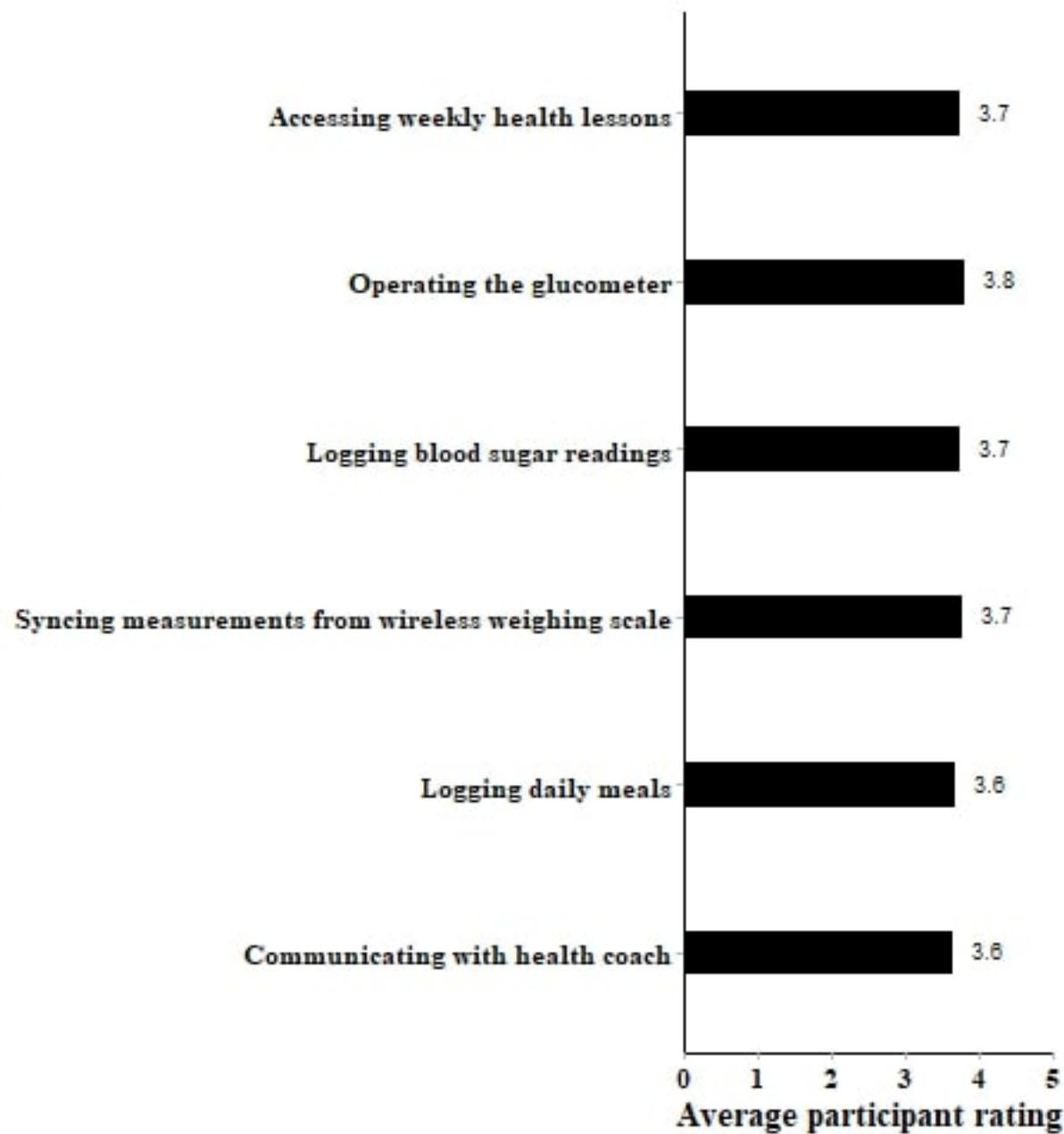

Supplement: Multimedia Appendix 9 [file mhealth_v7i5e12965_app9.pdf]

Program components

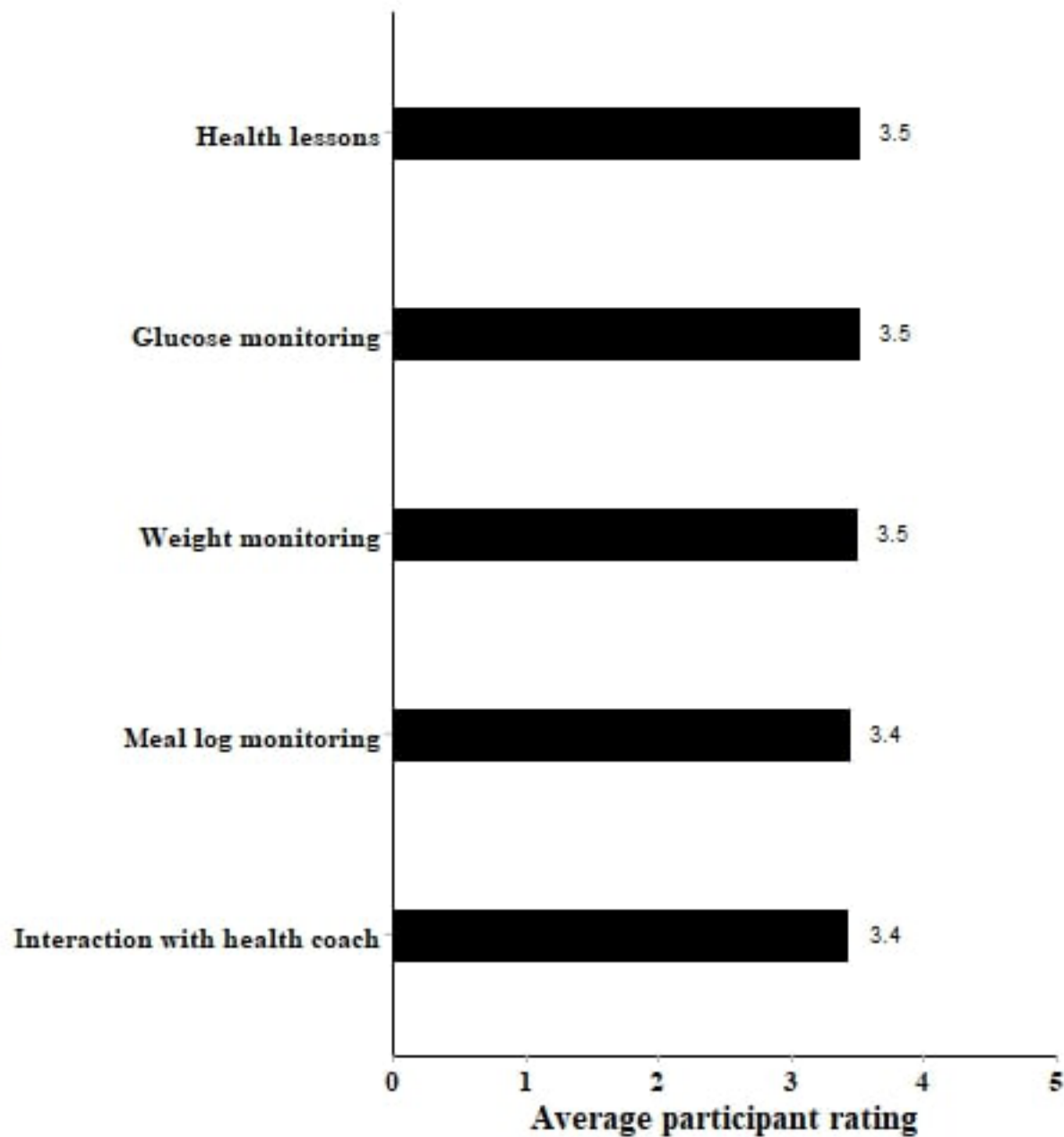

Supplement: Multimedia Appendix 10 [file mhealth_v7i5e12965_app10.pdf]

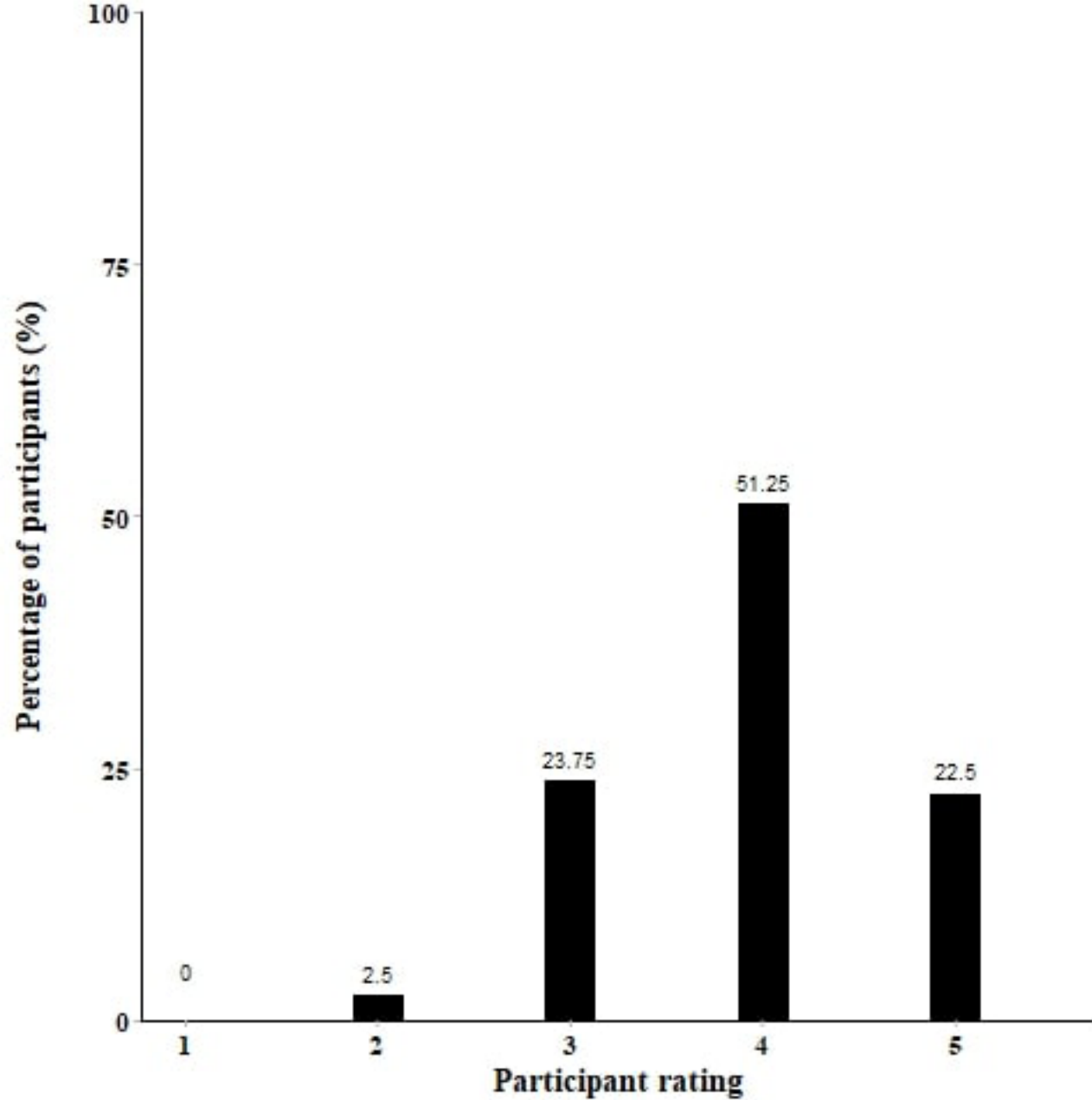

Supplement: Multimedia Appendix 11 [file mhealth_v7i5e12965_app11.pdf]
